# Circadian Patterns of Heart Rate Turbulence, Heart Rate Variability and Their Relationship

**DOI:** 10.4021/cr41w

**Published:** 2011-05-20

**Authors:** Hung Yi Chen

**Affiliations:** Department of Cardiology, Taipei City Hospital-Heping Branch, No. 33, Sec. 2, Zhonghua Rd., Taipei City 100, Taiwan. Email: anigi426@ms24.hinet.net; dae28@tpech.gov.tw

**Keywords:** Heart rate turbulence, Heart rate variability, Circadian variation

## Abstract

**Background:**

Heart rate variability (HRV) is an established tool for studying cardiac autonomic activity over time, while heart rate turbulence (HRT) is a recent method used to assess autonomic dysfunction. However, there are different autonomic tones at different times of a day. This study aimed to examine the effect of circadian change of autonomic tone on heart rate turbulence and variability, and determine any relationship between them based on circadian variations.

**Methods:**

Twenty-four-hour Holter recordings from 35 subjects with structurally normal heart were done, and HRV and HRT parameters of turbulence onset (TO) and turbulence slope (TS) were calculated. The 24-hour circadian patterns of these parameters and correlation analysis between them were performed.

**Results:**

There were conspicuous diurnal oscillations in TS and HRV parameters, with less prominent variation in TO. TS and high frequency power (HF) shared the similar oscillation patterns in a day. Comparing HRT and HRV based on circadian variation, TS showed positive correlations with HF and low frequency power (LF) to a reasonable degree, while there was an inverse correlation between TS and LF/HF.

**Conclusions:**

Circadian change of TS values throughout the day is more prominent than TO. It also presents as an autonomic activity more than TO. The relationships between HRV and HRT persist independently on the time of a day.

## Introduction

Decreased heart rate variability (HRV) that reflects an imbalance in cardiac autonomic tone is well documented [1]. Heart rate turbulence (HRT) has been introduced to represent the biphasic physiologic response of sinus rhythm to a single ventricular premature beat [2]. Variations consist of an initial acceleration and a subsequent sinus deceleration, which are believed to be mainly mediated by the cardiac autonomic nervous system. Several studies have shown that impaired HRT reflects cardiac autonomic dysfunction, particularly impaired baroreflex sensitivity and reduced parasympathetic activity [3-5]. However, there are different autonomic tones at different times of the day. This is evidenced by circadian changes in heart rate variability and heart rate turbulence parameters [6, 7]. To avoid the effect of autonomic oscillation in a day on heart rate turbulence and variability, this study aimed to evaluate diurnal autonomic cardio-vascular function in daily activity by analyzing the hourly values of heart rate variability and heart rate turbulence. The correlation between HRT and HRV was also evaluated based on circadian analysis.

## Methods

### Study subjects and data acquisition

All patients received 24-hour Holter ECG under the complain suspected of arrhythmia at the outpatient department for one year duration were collected. Those with ventricular arrhythmia as recorded by 24-hour Holter ECG were reviewed. Patients were included if they fulfilled the following criteria: (1) absence of known cardiovascular disease by history; (2) absence of significant structural heart disease by echocardiogram; (3) absence of medical history as diabetes mellitus, thyroid disorder, and liver or kidney disease; and (4) absence of any medication affecting heart rate/rhythm. The Holter ECG was recorded while the subjects went about their normal daily routines.

Ventricular premature ectopic beats were identified by a three-channel 24-hour Holter recorder via digitized Holter analyzer (Medilog FD4, Oxford Instruments) and analyzed using Medilog Excel-3 (Medilog Cardiology information system V2.3, Oxford Instruments). RR interval duration measurements and QRS morphology were automatically performed by a digitized Holter analyzer and obtained after review and manual editing by experienced staff.

### Heart rate turbulence

HRT was calculated as previously described [2]. Only isolated ventricular ectopic beats with a distinct post-ectopic pause were included in the HRT analysis. Two numerical descriptors were estimated: turbulence onset (TO), reflecting the initial phase of sinus rhythm acceleration, and turbulence slope (TS), describing the deceleration phase. Briefly, TO (%) was defined as the percentage difference between the mean of the first two RR intervals after a ventricular premature beat and the two last sinus RR intervals before a ventricular premature beat. TS was calculated as the maximum slope of the regression line over any sequence of five sinus RR intervals within the first 20 sinus beats after an ectopic beat.

Ventricular premature contractions were accepted if they had the following criteria: a coupling interval < 80% of the average of the preceding five sinus intervals; a compensatory pause > 120% of the preceding sinus intervals; and if they were embedded into two preceding and 20 succeeding N-N intervals. The N-N intervals also fulfilled the following criteria: cycle length > 300 ms but < 2000 ms; beat-to-beat difference < 200 ms; and difference to the reference interval < 20%. The HRT onset or slope was defined as abnormal if it was ≥ 0% or if the slope was ≤ 2.5 ms/beat.

HRT parameters were computed and obtained by a program written in MatLab software (version 6.1). While dividing the data into consecutive 24 hours, hourly values of TO and TS were calculated and obtained for each patient after averaging the total separated data in each hour. For analysis of circadian variation, subjects were excluded from the study if they had more than four unavailable hourly HRT data (without isolated VPC for HRT calculation). Only HRT data that could be evaluated in at least 20 separated hourly values were included.

### Heart rate variability

Heart rate variability analysis was assessed over a 24-hour period and was performed both in time and frequency domains according to the Task Force Guidelines of the ECS/NASPE [8]. RR intervals before and after the ectopic beats and intervals that varied by more than 20% were defined premature beats and excluded in the analysis, with a linear interpolated QRS based on the prior beat’s RR interval and the subsequent RR interval.

For analysis of circadian variation, both time and frequency domain HRV analyses were conducted from the 24 separated hourly recordings. For each recording, those with frequent premature atrial or ventricular beats with > 10% ectopic beats in each separated hour were excluded. Otherwise, the R time points were changed to reflect an interpolated time points. Time domain outcomes included the standard deviation of normal RR interval (SDNN), mean of the SD of all RR intervals for all five-minute segments (SDNNI), the square root of the mean of the sum of squares of differences between adjacent NN intervals (rMSSD), and the percentage of absolute differences between successive normal RR intervals that exceed 50 msec (pNN50). Frequency domain variables included the high-frequency power of 0.15 - 0.40 Hz (HF), low-frequency power of 0.04 - 0.15 Hz (LF), and very low-frequency power ≤ 0.04 Hz (VLF). The LF/HF ratio was also calculated. Beat-to-beat fluctuations were transformed to the frequency domain by fast Fourier Transformations. Only patients with at least 20 separated hourly values available were included for HRV analysis.

### Statistical analysis

Statistical analyses were conducted with a commercially available software package (SPSS, version 13.0; SPSS Inc.). All continuous variables were expressed as mean ± standard deviation and as median. To avoid skewed distributions, the quantities of VLF, LF, HF, LF/HF and rMSSD were log transformed before calculating the correlation coefficient. Correlations between HRT and HRV parameters were assessed using two-tailed Pearson’s correlation.

## Results

### Clinical characteristics of the study patients

The study evaluated 35 subjects (19 men and 22 women; age 21 - 61 years, mean age, 41 years). All showed normal left ventricular contractility (LVEF: 69.4 ± 5.1) and normal left heart chamber size (LVEDD: 46.5 ± 3.8 mm, and LA diameter: 30.4 ± 4.3 mm) by two-dimensional echocardiogram.

After the Holter recording was checked, there was a wide variation in the number of VPCs in different individuals, ranging from 108 to 7412 (mean ± SD: 2195 ± 1973) in 24 hours. HRT parameters showed a broad spectrum of values, with TO ranging from -14.25% to 12.16% and TS from 3 to 42.2 (ms/RR). When dichotomizing patients according to abnormal HRT using a cutoff value of TO > 0% and TS < 2.5 ms/RR as proposed previously [4], 26% (9/35) of subjects had abnormal TO while no subjects had TS < 2.5 ms/RR.

### Circadian profiles of HRT and HRV

**Figure 1:**
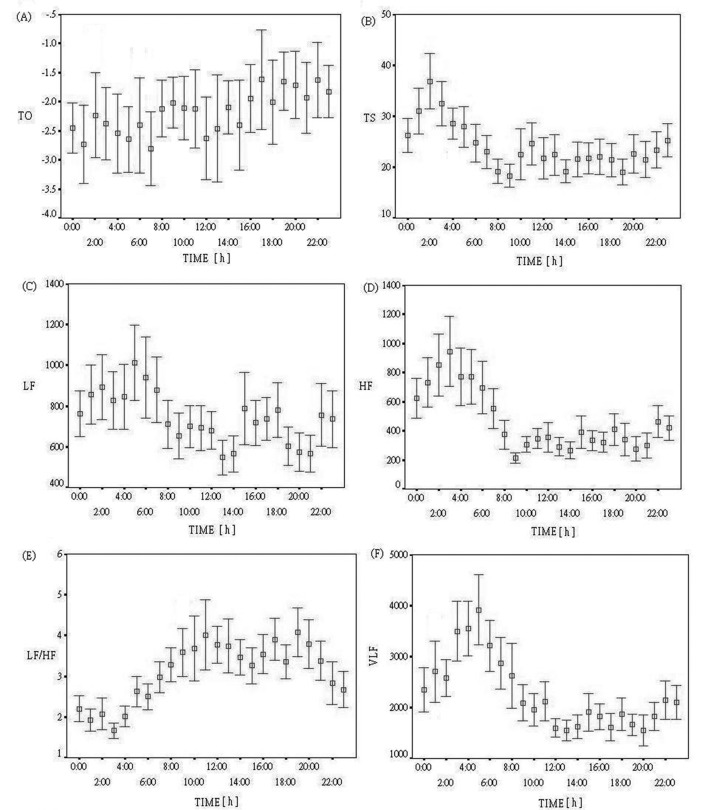

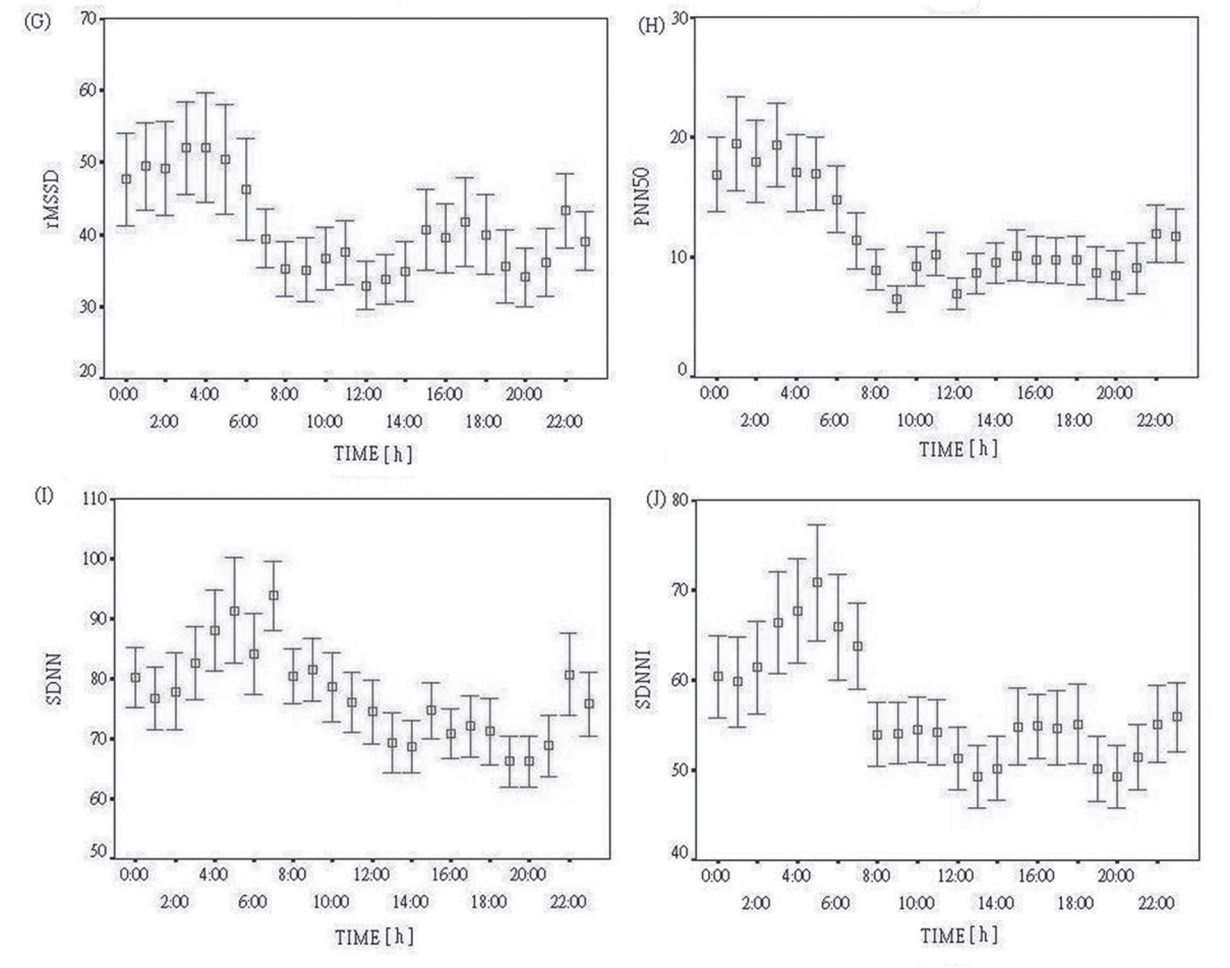
Hour-to-hour circadian variation (mean ± SD) of heart rate turbulence (HRT) and heart rate variability (HRV) parameters. (A) Turbulence onset (TO), (B) turbulence slope (TS), (C) low frequency (LF), (D) high frequency (HF), (E) low frequency/high frequency (LF/HF), (F) very low frequency (VLF), (G) square root of the mean of the sum of squares of differences between adjacent NN intervals (rMSSD), (H) percentage of absolute differences between successive normal RR intervals that exceed 50 msec (pNN50), (I) standard deviation of normal RR interval (SDNN), and (J) mean of the SD of all RR intervals for all five minute segments (SDNNI).

Analyzing all patients together, for heart turbulence, TS values were higher at nighttime than during daytime. However, circadian change was less prominent in TO than in TS values, while TO values at night were slightly lower than during the day. Analyzing circadian variation in heart rate variability, there were similar changes during nighttime sleep in time domain measurements (SDNN, SDNNI, pNN50 and rMSSD) and in frequency domain (VLF, LF and HF). All of these HRV parameters had higher values at nighttime than during daytime. In contrast, LF/HF values decreased during nighttime sleep. Circadian variation of the mean values of HRT (TO, TS) and HRV parameters (VLF, LF, HF, LF/HF, pMM50, rMSSD, SDNN, and SDNNI) were shown in Figure 1.

### Correlation between HRT and HRV data

**Figure 2:**
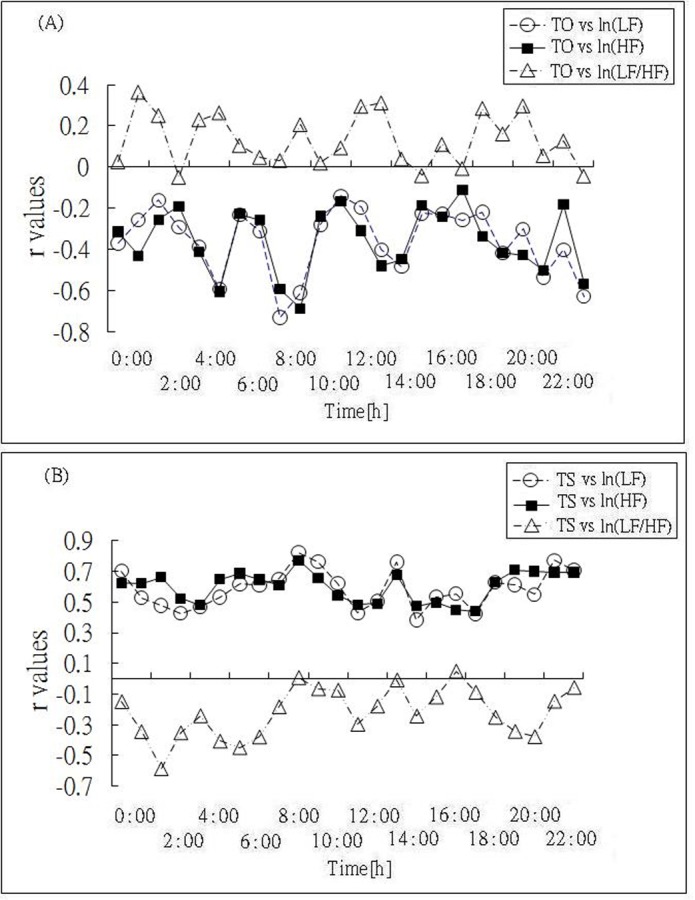
Time courses of the correlation coefficient r of the relationship between HRV frequency domains ln(LF), ln(HF), and ln(LF/HF) and (A) TO and (B) TS values.

When the relationship between HRT and HRV in each 24 hourly recordings was analyzed, the results showed a positive correlation to a reasonable degree in frequency domain between TS versus LF and HF values, whereas TS and LF/HF were negatively correlated, while the correlation coefficient r of the relationship TS versus LF/HF was stronger during nighttime. In contrast, TO versus LF, and HF indicated weak negative correlation although there was positive correlation between TO values and LF/HF. In addition, the correlation coefficient r of the relationship between TO versus HRV frequency domain data was weaker than that of TS versus HRV frequency domain in 24 separated hourly durations. Calculations of correlation coefficient between HRT and HRV frequency domain were showed in Figure 2.

**Figure 3:**
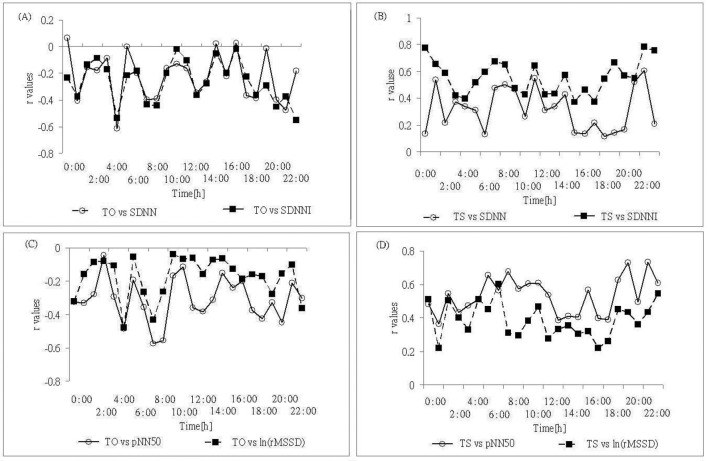
Time courses of the correlation coefficient r of the relationship between HRV time domain and HRT values. (Upper panel) SDNN, SDNNI vs. (A) TO and (B) TS values; and (Lower panel) pNN50, rMSSD vs. (C) TO and (D) TS values.

Regarding TS versus time-domain analysis of HRV, there were fairly positive correlations between TS versus pNN50 and SDNNI, and weak positive correlations with respect to rMSSD and SDNN. On the other hand, there was negative correlation between TO and HRV time domain. Calculations of correlation coefficients between HRT and HRV time-domains were demonstrated in Figure 3.

## Discussion

Both HRV and HRT are methods for evaluating autonomic activity of the heart. The aim of this study is to clarify the basic relationship between measurements of HRV and HRT during daytime activities and nighttime sleep. Most studies involving these two have used 24-hour Holter recorded electrocardiograms. However, circadian variations of autonomic tone are well known. The most rapid increase in sympathetic effect and decrease in vagal-tone in the early morning hours in normal individual are related to cardiovascular events.

Nonetheless, there are oscillations of autonomic tone at different times of the day. A value for HRV and HRT at a certain time of the day does not fully reflect autonomic activity in other times and in different conditions in a 24-hour period. Under this premise, the 24-hour Holter ECG has been divided into 24 consecutive hourly periods for HRT and HRV calculations. As such, parameters of each patient in 24 separated one-hour periods are calculated and obtained, thereby clarifying the basic relationships between HRV and HRT in a day.

With such assessments, the TS values and HRV parameters show prominent variations by different hours in a day. However, the diurnal variation curves in TO are more flat than those of TS values. These are consistent with results in a previous study [7]. Cygankiewicz et al. separated 24-hour into 3 periods, and after calculating HRT values, they demonstrated that TS values were lower in the afternoon (4 - 8 p.m.) than at night (0 - 4 a.m.) or morning hours (6 - 10 a.m.). There is no significant difference between night and morning hours. Unlike their report, the current study shows that TS values are higher during sleep times than in the morning and afternoon hours, although the differences between morning and afternoon hours are not prominent. Moreover, we found there was a dip of TS values in the morning (8 - 9 a.m.) calculated by hourly values in this study.

There are different characteristic patterns in circadian profiles of HRV and TS values in this study. The most important finding is that there are similar diurnal oscillation patterns in TS and HF values. Both increase with steep curves during sleep and peak at around 2 - 3 a.m. After that, both demonstrate progressively declining curves, with a dip in the morning hours (8 - 9 a.m.). The circadian patterns of VLF and LF also increase during sleep (nighttime) but peak around 5 a.m. As for LF/HF, there is an inverse circadian pattern with a minimum around 3 a.m. On the other hand, time domain rMSSD and pNN50 also reaches a maximum at around 2 - 4 a.m., but with less rapid upslope than TS and HF during the initial sleep times. Like LF, VLF, time domain SDNN, and SDNNI all had maximal values around 5 - 6 a.m. Thus, the hour of the maximal TS, HF, rMSSD, and pNN50 values is similar, 2 to 3 hours prior to the hour of peak VLF, LF, SDNN, and SDNNI values. Furthermore, TS and HF share similar curve by hour-to-hour analysis.

The relationship between HRT and HRV can be quantified by their correlation coefficient, which is another important finding in this study. Regardless of the time of day, values of the correlation coefficient r between TS and LF, HF remain stable to a reasonable degree. There is also fairly negative correlation between TS and LF/HF.

Heart rate and heart rate variability depend on the influence of sympathetic and parasympathetic tone on the sinus node and reflect spontaneous changes in autonomic tone. Vagal tone is expressed by HF and the sympatho-parasympathetic balance, by the LF/HF ratio, although their interpretation is sometimes controversial. Possible reasons may be related to different mechanisms in different conditions and to the interaction between heart rate and other biological signals. For HRT, the real physiologic mechanism is not well established. It reflects baroreflex but may be a measure of autonomic response of arterial blood invoked by VPCs. A previous study shows that HRT is diminished by atropine, or atropine plus esmolol, but not by esmolol alone [5].

There are some reports showing that impaired HRT reflects cardiac autonomic dysfunction and reduces parasympathetic activity [3, 4]. Another study reveals that HRT is mainly mediated by the vagus nerve and that sympathetic blockade has no influence on HRT parameters [9]. These previous studies suggest the relationship between HRT and parasympathetic activity. Whether the late deceleration phase of HRT involves sympathetic or vagal activity remains unclear and debatable. The results here hint that TS is probably the result of a complex interaction of both systems, as posited by some previous reports [10]. Furthermore, it might be mainly mediated by vagal tone. And the relationships between HRV and HRT persist independently on the time of a day.

### Limitations

All of the subjects went about their daily routines. Different activities and different conditions in a day in different individuals are distinct. This could have been attenuated by a design that compared each subject’s hourly own data of HRV and HRT parameters. Moreover, since the inter-individual differences of daily activities are large, for example there are different hourly times of work and rest in each subject, this may influence the circadian patterns of HRV and HRT by hourly time.

Another disadvantage is the relatively small sample size. Since VPCs are necessary in HRT calculations, subjects without adequate VPC in 24 separated hourly durations have been excluded. In HRV calculations, patients with too frequent extra beats have also been excluded to avoid the impact on HRV parameters. The inclusion criteria for HRT and HRV in the study protocols may also be conflicting. This limited the sample size, thereby possibly influencing the results.

### Conclusions

Turbulence slope and most of the HRV parameters increase during sleep times and fully reflect oscillation of autonomic tone in a day. On the other hand, both the TO and LF/HF values decline during sleeping times. Moreover, there is less prominent oscillation in TO values during the day than other HRT and HRV parameters. Comparing HRT and HRV parameters by circadian variation, regardless of the time in a day, there is a correlation between TS values and HRV frequent domain LF and HF, particularly in terms of similar circadian patterns.
